# Author Correction: The mitochondrial β-oxidation enzyme HADHA restrains hepatic glucagon response by promoting β-hydroxybutyrate production

**DOI:** 10.1038/s41467-026-75461-3

**Published:** 2026-07-09

**Authors:** An Pan, Xiao-Meng Sun, Feng-Qing Huang, Jin-Feng Liu, Yuan-Yuan Cai, Xin Wu, Raphael N. Alolga, Ping Li, Bao-Lin Liu, Qun Liu, Lian-Wen Qi

**Affiliations:** 1 State Key Laboratory of Natural Medicines, School of Traditional Chinese Pharmacy, China Pharmaceutical University, Nanjing, 210009 China; 2 Clinical Metabolomics Center, China Pharmaceutical University, Nanjing, 211198 China

**Keywords:** Type 2 diabetes, Metabolic syndrome, Diabetes

Correction to: *Nature Communications* 10.1038/s41467-022-28044-x, published online 19 January 2022

In the version of this article initially published, due to a data transfer error, the Source Data and plot of Fig. 1c were incorrect, while Source Data for Figs. 3g, 6a, 7h had duplication errors and Supplementary Fig. 3a had missing values. In Fig. 4b, representative images in the third column were duplicates of the third column of Fig. 4c. The figures and Source Data are now amended in the HTML and PDF versions of the article.

