# Supplementary material for: The mitochondrial β-oxidation enzyme HADHA restrains hepatic glucagon response by promoting β-hydroxybutyrate production
Source: Nat Commun. 2022 Jan 19;13:386. doi: 10.1038/s41467-022-28044-x (PMC8770464; doi:10.1038/s41467-022-28044-x)
Supplement: Supplementary file 1 — Supplementary Information [file 41467_2022_28044_MOESM1_ESM.pdf]

Supplementary Information

The mitochondrial β-oxidation enzyme HADHA restrains hepatic glucagon response by promoting β-hydroxybutyrate production

Pan et al.

Supplementary Materials and Methods

Quantitative real-time reverse transcription PCR (qRT-PCR). The total mRNA from different tissues and primary hepatocytes were isolated using TRIzol™ Reagent (Invitrogen, America) to synthesize cDNAs. qRT-PCR was performed on the Roche LightCycler 96 System using the Fast SYBR Green Master Mix (Roche, America). The mRNA expression levels of target genes were normalized to *Actb* expression levels.

In vitro transfection. For HADHA, BDH1 and HDAC7 knockdown, primary hepatocytes were transfected with siRNA using Lipofectamine® 2000 transfection reagent (ThermoFisher, America). The siRNA oligos are as follows:

Normal control (NC) siRNA: UUCUCCGAACGUGUCACGUTT

ACGUGACACGUUCGGAGAATT

HADHA siRNA 1: GGUUGCCAUUUCAUGCCAATT

UUGGCAUGAAAUGGCAACCTT

HADHA siRNA 2: CCAAAGGACUAGCUGAUAATT

UUAUCAGCUAGUCCUUUGGTT

HADHA siRNA 3: CCGCCUGGUGACAAGAUUUTT

AAAUCUUGUCACCAGGCGGTT

BDH1 siRNA 1: GCAGGAAGUACUUCGAUGAAA

UUUCAUCGAAGUACUUCCUGC

BDH1 siRNA 2: GAUUGCCAAGAUGGAAACCUA

UAGGUUUCCAUCUUGGCAAUC

BDH1 siRNA 3: GAUGCAGAUCAUGACCCAUUU

AAAUGGGUCAUGAUCUGCAUC

HDAC7 siRNA 1: GCAGUGUGGUCAAGCAGAA

UUCUGCUUGACCACACUGC

HDAC7 siRNA 2: GGCAGGCUUACACCAGCAA

UUGCUGGUGUAAGCCUGCC

HDAC7 siRNA 3: CCUGAAGUUGCGCUACAAA

UUUGUAGCGCAACUUCAGG

Animals and treatments. All animal treatments were approved by the Animal Ethics Committee of China Pharmaceutical University (protocol no. 2019-04-002). Animal testing and research conformed to all relevant ethical regulations. Six to eight-week-old male C57BL/6J mice were purchased from Nanjing Junke Biotechnology Co, Ltd. (China). After a week of acclimation, the mice were raised in a temperature-controlled facility on a 12 h light-dark cycle with free access to food and water. For high-fat diet (HFD) feeding, mice were fed with HFD (60% kcal from fat; D12492; Research diet, America) or normal chow diet (10% kcal from fat; Xietong Organism, China) for 12 weeks.

For glucagon challenge experiments, the mice were intraperitoneally injected with 2 mg/kg glucagon (1 h, Novo Nordisk, Denmark). BHB (100 mg/kg, Aladdin, China) or somatostatin (3 mg/kg, Merck Serono, Switzerland) were administrated to mice by intraperitoneal injection 1 h or 15 min before glucagon challenge, respectively.

Stable isotope tracing of acetyl-CoA by liquid chromatography coupled with triple quadrupole mass spectrometer (LC-MS/MS). For stable isotope tracing, the [U-^{13}C]palmitate was conjugated with phosphate buffer saline (PBS) containing 6.02 mM bovine serum albumin (BSA). Primary hepatocytes were transfected with HADHA plasmid for 24 h and treated with [U-^{13}C]palmitate (0.1 mM) for 4 h. For the extraction of acetyl-CoA, primary hepatocytes were first washed once with ice-cold PBS to remove the medium components and subsequently rinsed with water. Then the cells were ultrasound-crushed in 1 mL of methanol for 10 min. After centrifugation (12,000 x g, 4 °C, 10 min), 900 μL of supernatants were carefully transferred and dried by a gentle stream of nitrogen gas. Finally, the residue was reconstituted with 100 μL of 50% methanol aqueous solution, and 2 μL injection was analyzed by mass spectrometry.

Targeted detection was performed on a LC-20A system coupled to a triple quadrupole mass spectrometer (Shimadzu, LC-MS/MS 8050) operating in the negative ion mode. The chromatographic separation was achieved on a reversed-phase C18 column (Waters, 2.1×100 mm, 1.8 μm) maintained at 40 °C at a flow rate of 0.2 mL/min. The mobile phase consisted of water with 5 mM ammonium formate (A) and

acetonitrile (B). The gradient elution program was 5-20% B at 0-5 min, 20-95% B at 5-8 min, 95% B at 8-10 min, and then back to initial conditions, with 2 min for equilibration. The ESI source parameters were set as follows: DL temperature, 250 °C; interface temperature, 250 °C; heat block temperature, 400 °C; heating gas flow, 10 L/min; nebulizing gas flow, 3 L/min; and drying gas flow, 10 L/min. Multiple reaction monitoring mode was applied and the detailed ion transitions were: acetyl-CoA, *m/z* 810→303 (M), *m/z* 812→305 (M+2). Multiple reaction monitoring chromatograms were provided in Supplementary Data 1.

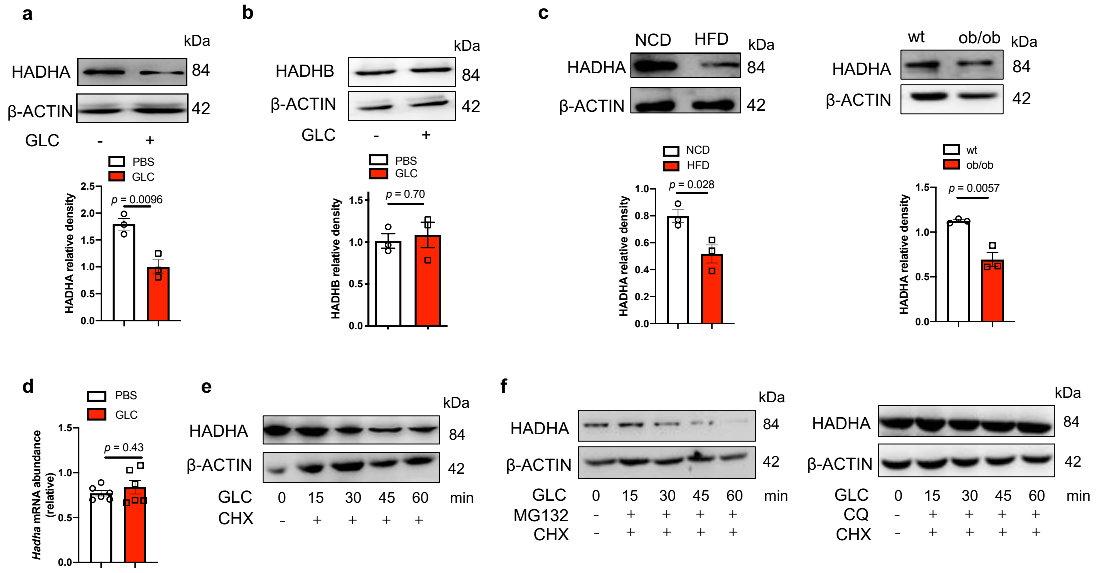

Supplementary Fig. 1 Glucagon impaired HADHA protein stability via lysosomal degradation. a Western blotting analysis of HADHA expression in primary hepatocytes treated with glucagon (100 nM, 1 h, n = 3). **b** Western blotting analysis of HADHB expression in primary hepatocytes treated with glucagon for 1 h (100 nM, n = 3). **c** Hepatic HADHA protein level in HFD-fed mice (n = 3) or ob/ob mice (n = 3). **d** Relative mRNA abundance of *Hadha* in glucagon-stimulated (100 nM, 1 h) primary hepatocytes (n = 6). **e** Western blotting analysis of HADHA in glucagon-stimulated (100 nM, 1 h) primary hepatocytes treated with CHX (20 μM). It was repeated 3 times independently with similar results. **f** HADHA protein level in glucagon-stimulated (100 nM, 1 h) primary hepatocytes treated with MG132 (20 μM) or CQ (20 μM). It was repeated 3 times independently with similar results. CHX cycloheximide, CQ chloroquine, GLC glucagon, HFD high-fat diet, NCD normal chow diet, PBS phosphate buffer solution, wt wild type. Values represent mean ± SEM. Statistical differences were determined by a two-tailed Student's *t* test. Source data are provided as a Source Data file.

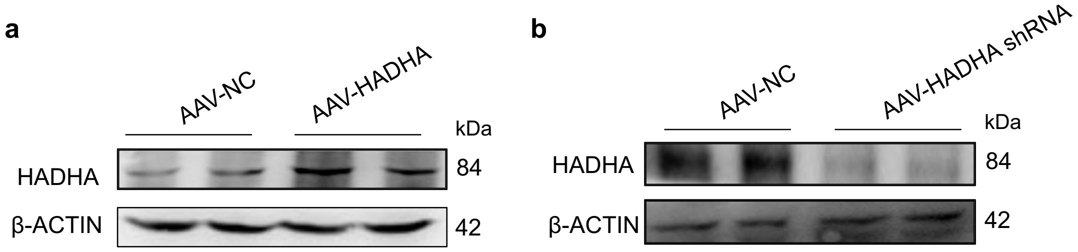

Supplementary Fig. 2 Western blotting confirmed HADHA protein expression. a Western blotting analysis of HADHA in the liver of mice injected with AAV8-HADHA or AAV8-NC. **b** Hepatic HADHA expression of mice injected with AAV8-HADHA shRNA or AAV8-NC. They were repeated 3 times independently with similar results. AAV adeno-associated virus, NC normal control.

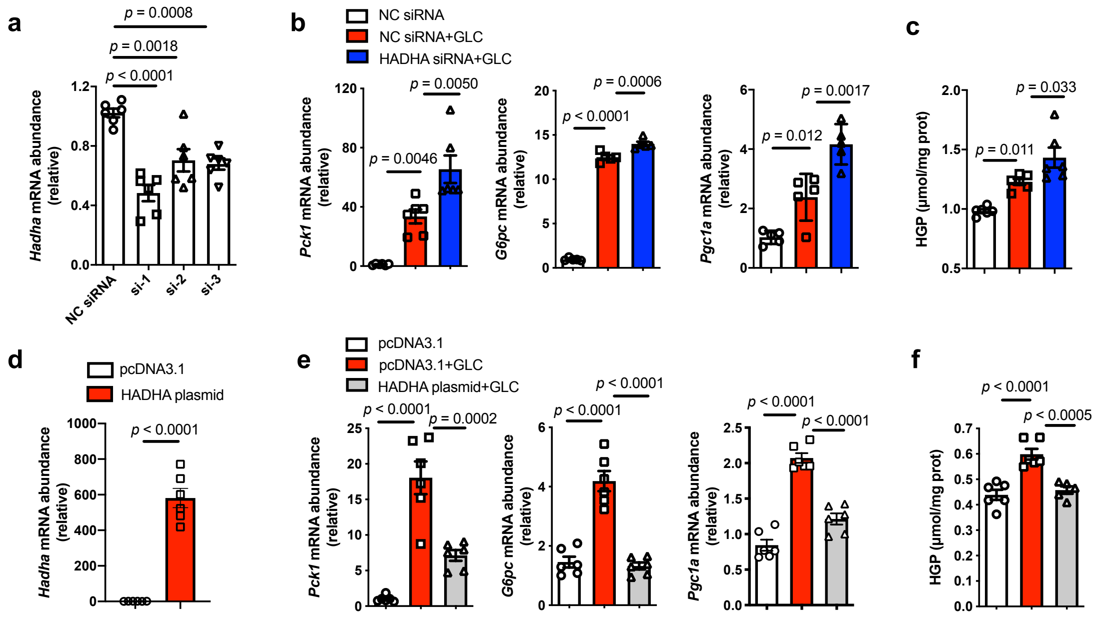

Supplementary Fig. 3 HADHA inhibited hepatic glucagon response in vitro. a mRNA levels of *Hadha* siRNA sequences in primary hepatocytes (n = 6). **b** Relative mRNA abundance of *Pck1*, *G6pc* and *Pgc1a* in primary hepatocytes stimulated by glucagon (100 nM, 1 h) after HADHA knockdown (n = 6). **c** HGP from primary hepatocytes in panel **b** (n = 6). **d** mRNA levels of *Hadha* in HADHA-overexpressed primary hepatocytes (n = 6). **e** Relative mRNA abundance of *Pck1*, *G6pc* and *Pgc1a* in primary hepatocytes stimulated by glucagon (100 nM, 1 h) after HADHA overexpression (n = 6). **f** HGP from primary hepatocytes in panel **e** (n = 6). AAV adeno-associated virus, GLC glucagon, HGP hepatic glucose production, NC normal control. Bars represent mean ± SEM values. Statistical differences between two groups were determined by a two-tailed Student's *t* test, and all others were used by one-way ANOVA. Source data are provided as a Source Data file.

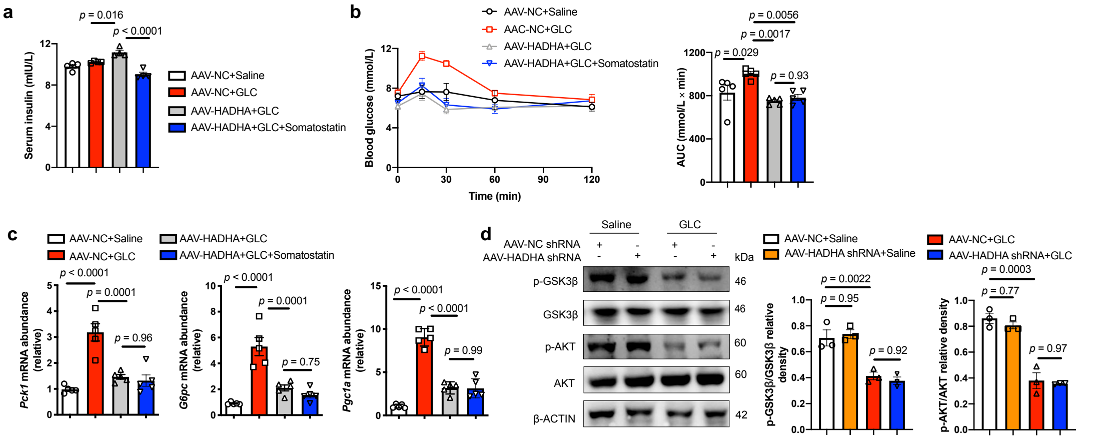

Supplementary Fig. 4 HADHA inhibited hepatic glucagon response independently of insulin release. a Serum insulin levels of the mice treated with AAV8-HADHA or somatostatin (3 mg/kg, 15 min), glucagon stimulation for 1 h (n = 5). **b** Blood glucose levels in normal mice subjected to glucagon challenge (2 mg/kg, 1 h) and treated with AAV8-HADHA or somatostatin (3 mg/kg, 15 min). AUC is indicated on the right (n = 5). **c** mRNA levels of *Pck1*, *G6pc* and *Pg1a* in the livers of the mice in panel **b** (n = 5). **d** Western blotting analysis of p-GSK3β, GSK3β, p-AKT and AKT in the liver of mice injected with AAV8-HADHA shRNA or AAV8-NC in the absence or presence of glucagon (2 mg/kg, 1 h, n = 3). AAV adeno-associated virus, AUC area under the curve, GLC glucagon, NC normal control, Bars represent mean ± SEM values. Statistical differences were determined by one-way ANOVA. Source data are provided as a Source Data file.

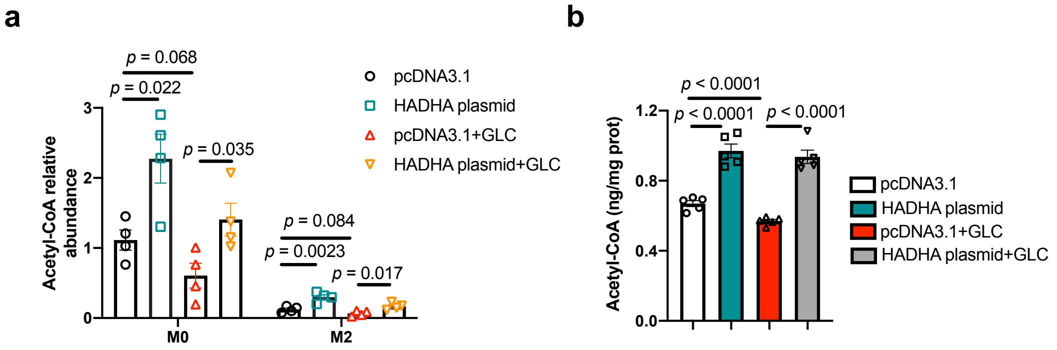

Supplementary Fig. 5 HADHA promoted acetyl-CoA production. a Relative acetyl-CoA level in primary hepatocytes treated with [U-^{13}C]palmitate (0.1 mM) for 4 h followed by 100 nM glucagon stimulation for 1 h with or without HADHA plasmid transfection (n = 4). **b** Acetyl-CoA levels in HADHA-overexpressed primary hepatocytes with or without 100 nM glucagon stimulation for 1 h (n = 5). GLC glucagon. Bars represent mean ± SEM values. Statistical differences were determined by one-way ANOVA. Source data are provided as a Source Data file.

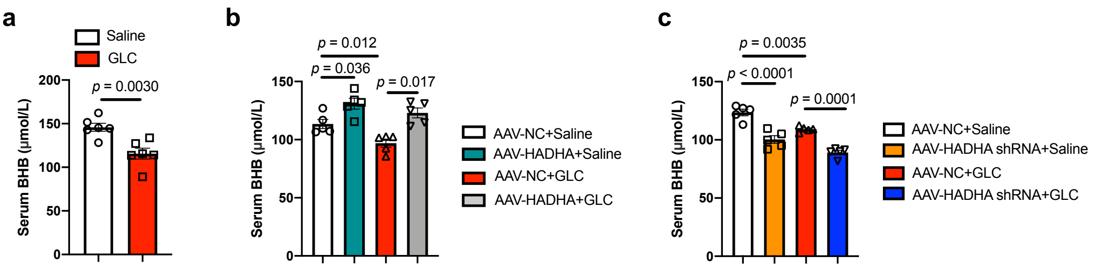

Supplementary Fig. 6 HADHA promoted BHB production in mice. a Serum BHB levels in glucagon challenge mice (2 mg/kg, n = 6). **b, c** Serum BHB levels in HADHA liver-specific overexpression (**b**) or knockdown (**c**) mice with or without glucagon stimulation (2 mg/kg, n = 5). AAV adeno-associated virus, BHB β-hydroxybutyrate, GLC glucagon, NC normal control. Values represent mean ± SEM. Statistical differences between two groups were determined by a two-tailed Student's *t* test, and all others were used by one-way ANOVA. Source data are provided as a Source Data file.

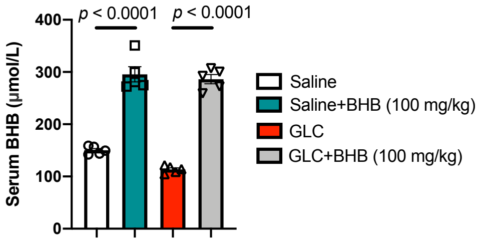

Supplementary Fig. 7 The serum level of BHB was elevated after BHB administration in mice. BHB (100 mg/kg) was administrated with or without glucagon (2 mg/kg, 1 h) in mice (n = 5). BHB β-hydroxybutyrate, GLC glucagon. Values represent mean ± SEM. Statistical differences were determined by two-tailed Student's *t* test. Source data are provided as a Source Data file.

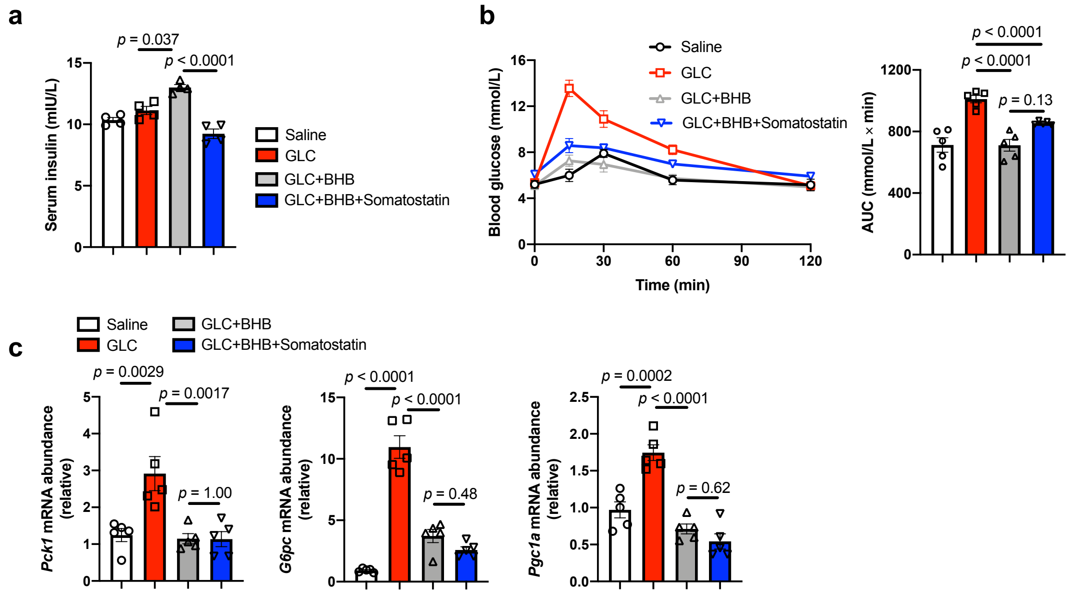

Supplementary Fig. 8 BHB inhibited glucagon response independently of insulin release. a Serum insulin levels in the mice subjected to glucagon challenge (2 mg/kg), treatment with BHB (100 mg/kg) and somatostatin (3 mg/kg, 15 min, n = 4). **b** Blood glucose levels in normal mice subjected to glucagon challenge (2 mg/kg), treatment with BHB (100 mg/kg) and somatostatin (3 mg/kg, 15 min). AUC is indicated on the right (n = 5). **c** mRNA levels of *Pck1*, *G6pc*, and *Pgc1a* in the livers of the mice in panel **b** (n = 5). AUC area under the curve, BHB β-hydroxybutyrate, GLC glucagon, NC normal control. Bars represent mean ± SEM values. Statistical differences were determined by one-way ANOVA. Source data are provided as a Source Data file.

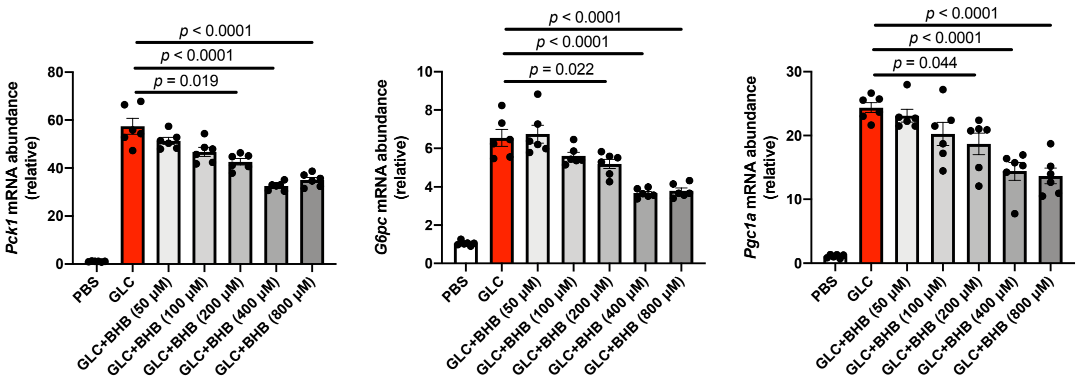

Supplementary Fig. 9 BHB inhibited gluconeogenesis in a dose-dependent manner. Relative mRNA abundance of *Pck1*, *G6pc* and *Pgc1a* in glucagon-stimulated (100 nM, 1 h) primary hepatocytes with or without BHB treatment (50, 100, 200, 400, and 800 μM, n = 6). BHB β-hydroxybutyrate, GLC glucagon, PBS phosphate buffer solution. Bars represent mean ± SEM values. Statistical differences were determined by one-way ANOVA. Source data are provided as a Source Data file.

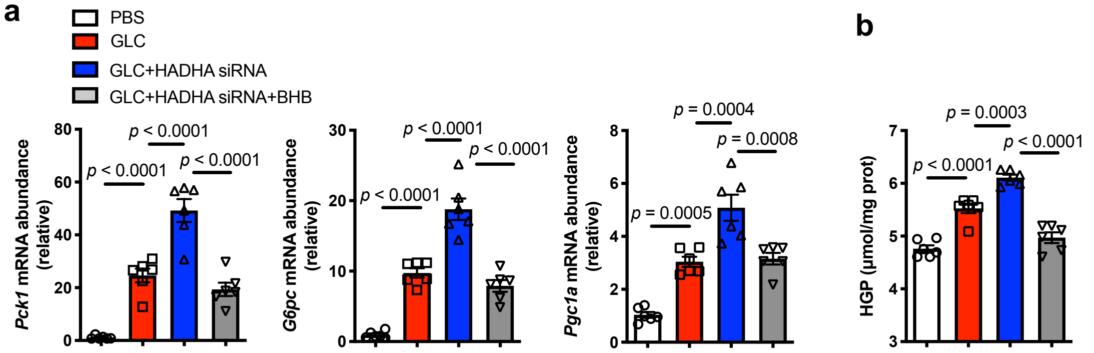

Supplementary Fig. 10 Exogenous BHB antagonized HADHA knockdown-enhanced gluconeogenesis in primary hepatocytes. a Relative mRNA abundance of *Pck1*, *G6pc*, and *Pgc1a* in HADHA-knockdown primary hepatocytes with or without BHB (400 μM, 6 h), glucagon (100 nM) stimulation for 1 h (n = 6). **b** HGP from primary hepatocytes in panel **a** (n = 6). BHB β-hydroxybutyrate, GLC glucagon, HGP hepatic glucose production, PBS phosphate buffer solution. Bars represent mean ± SEM values. Statistical differences were determined by one-way ANOVA. Source data are provided as a Source Data file.

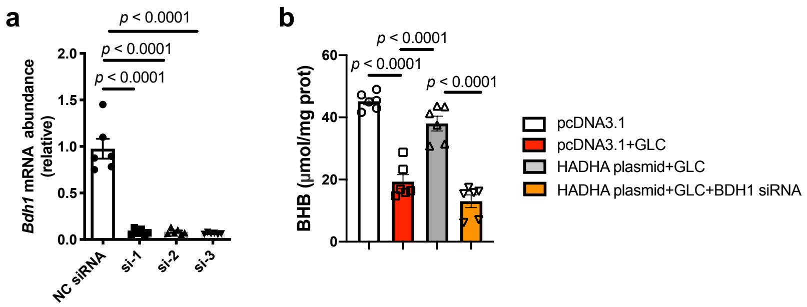

Supplementary Fig. 11 Silencing BDH1 antagonized HADHA overexpression-induced BHB production in hepatocytes. a mRNA levels of *Bdh1* siRNA sequences in primary hepatocytes (n = 6). **b** BHB level in HADHA-overexpressed primary hepatocytes transfected with BDH1 siRNA and 100 nM glucagon stimulation for 1 h (n = 6). BHB β-hydroxybutyrate, GLC glucagon, NC normal control. Bars represent mean ± SEM values. Statistical differences were determined by one-way ANOVA. Source data are provided as a Source Data file.

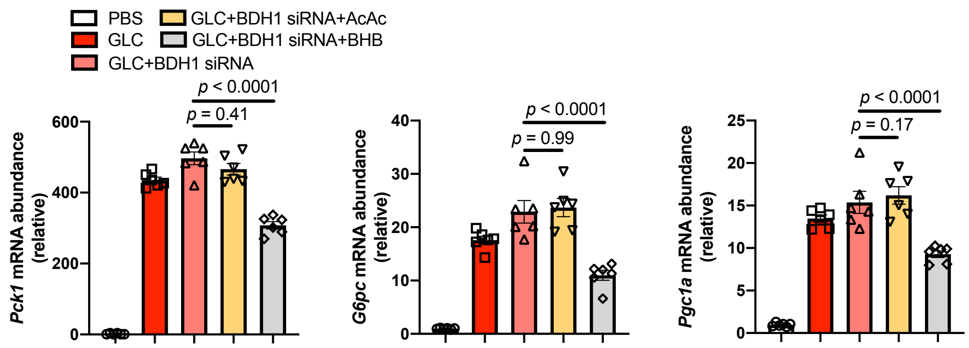

Supplementary Fig. 12 BHB, but not AcAc inhibited gluconeogenesis. Relative mRNA abundance of *Pck1*, *G6pc* and *Pgc1a* in primary hepatocytes transfected with BDH1 siRNA in the presence of BHB or AcAc (400 µM, 6 h) and 100 nM glucagon stimulation for 1 h (n = 6). AcAc acetoacetate, BHB β-hydroxybutyrate, GLC glucagon, PBS phosphate buffer solution. Bars represent mean ± SEM values. Statistical differences were determined by one-way ANOVA. Source data are provided as a Source Data file.

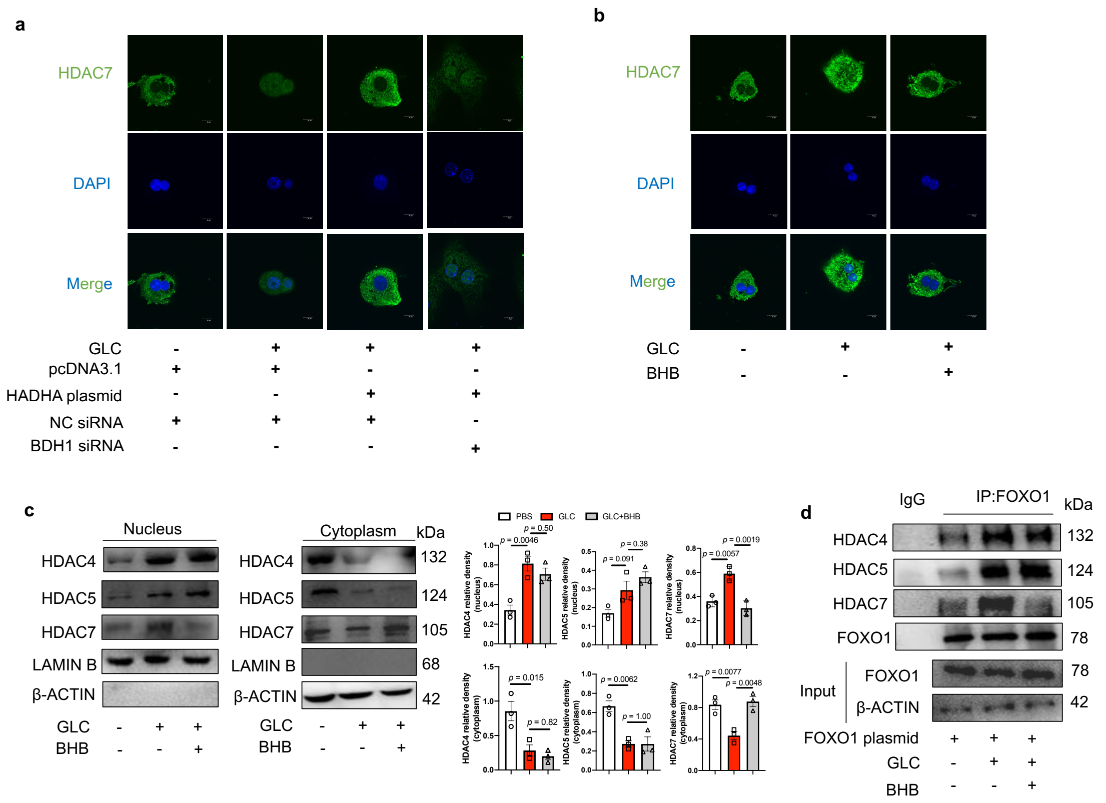

Supplementary Fig. 13 BHB selectively inhibited HDAC7 nucleus translocation and its interaction with FOXO1. a Representative confocal image of primary hepatocytes transfected with HADHA plasmid with or without BDH1 siRNA, 100 nM glucagon stimulation for 1 h. **b** Confocal images of glucagon-stimulated (100 nM, 1 h) primary hepatocytes with or without BHB (400 µM, 6 h). Scale bar represents 10 µm. **c** Western blotting analysis of nucleus and cytoplasm HDAC4, HDAC5 and HDAC7 expressions in glucagon-challenged (100 nM, 1 h) hepatocytes with or without BHB addition (400 µM, 6 h, n = 3). **d** Immunoprecipitation analysis of the interaction of FOXO1 with HDAC4, HDAC5 or HDAC7 in hepatocytes in glucagon-challenged (100 nM, 1 h) hepatocytes with or without BHB addition (400 µM, 6 h). **a**, **b** and **d** were repeated 3 times independently with similar results. BHB β-hydroxybutyrate, GLC glucagon, PBS phosphate buffer solution, NC normal control. Values represent mean ± SEM. Statistical differences were determined by one-way ANOVA. Source data are provided as a Source Data file.

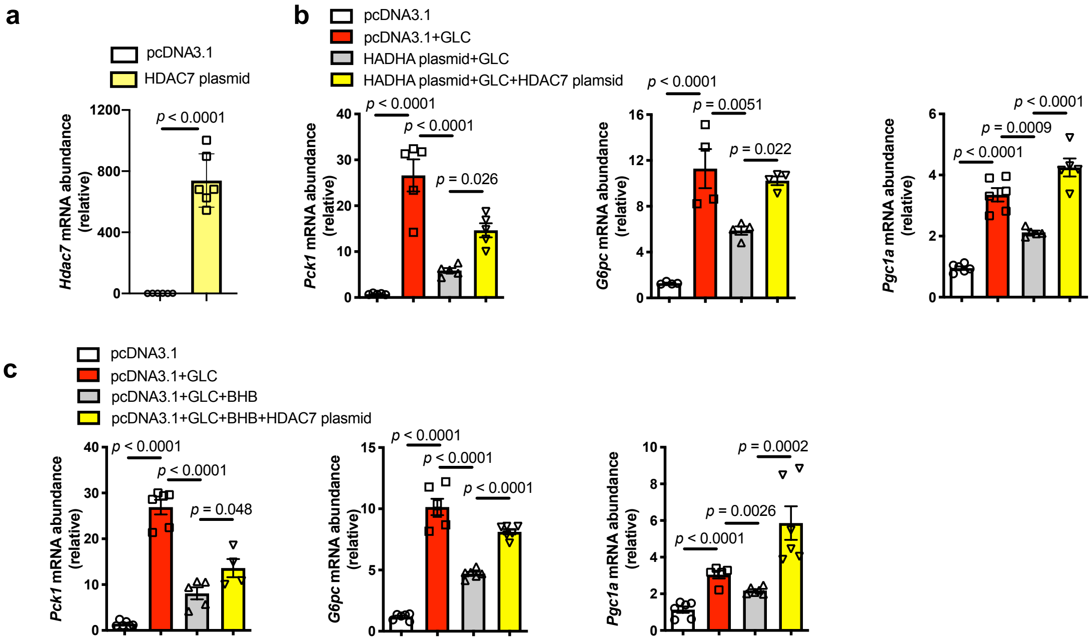

Supplementary Fig. 14 HDAC7 overexpression diminished the inhibitory effects of HADHA or BHB on glucagon-induced gluconeogenesis. a mRNA levels of *Hdac7* in HDAC7 overexpressed primary hepatocytes (n = 6). **b** Relative mRNA abundance of *Pck1*, *G6pc* and *Pgc1a* in HADHA-overexpressed primary hepatocytes with or without HDAC7 plasmid transfection, 100 nM glucagon stimulation for 1 h (n = 5). **c** Relative mRNA abundance of *Pck1*, *G6pc* and *Pgc1a* in BHB-treated (400 μM, 6 h) hepatocytes with or without HDAC7 plasmid transfection (n = 5). BHB β-hydroxybutyrate, GLC glucagon, NC normal control. The data are presented as the mean ± SEM. Statistical differences between two groups were determined by a two-tailed Student's *t* test, and all others were used by one-way ANOVA. Source data are provided as a Source Data file.

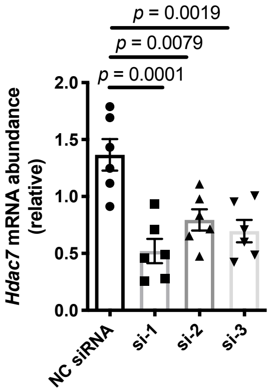

Supplementary Fig. 15 siRNA successfully inhibited *Hdac7* gene transcription. mRNA levels of HDAC7 siRNA sequences in primary hepatocytes (n = 6). NC normal control. The data are presented as the mean ± SEM. Statistical differences were determined by one-way ANOVA. Source data are provided as a Source Data file.

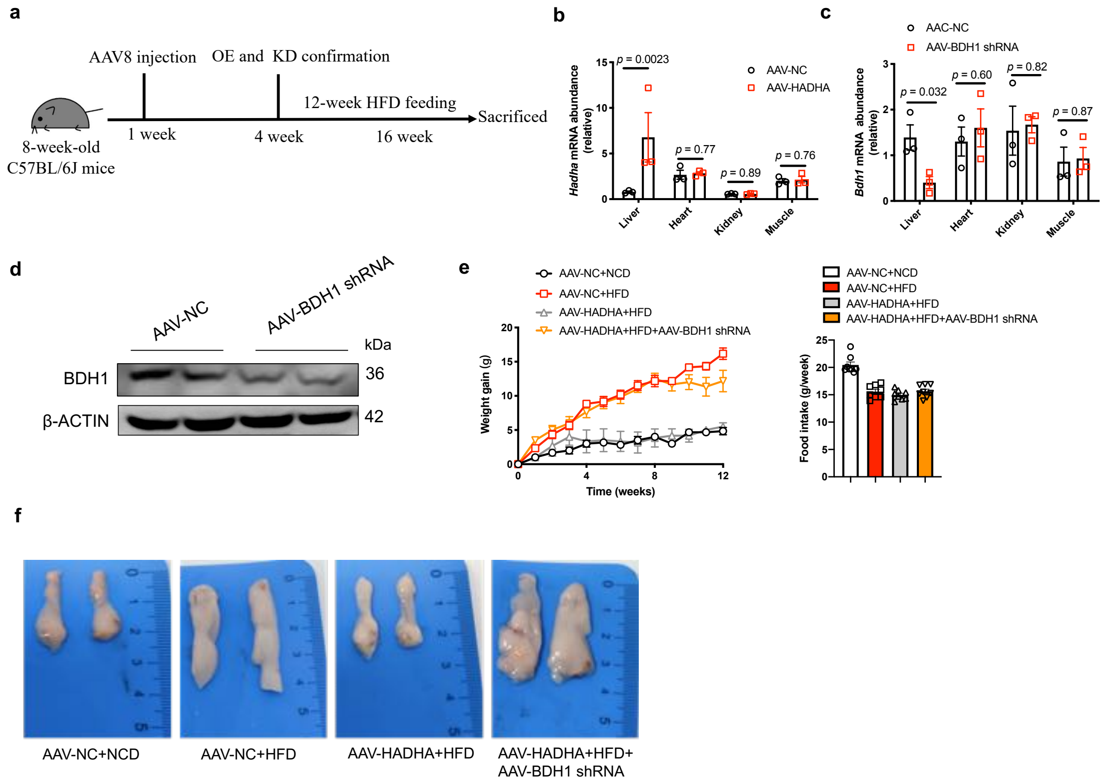

Supplementary Fig. 16 Hepatic HADHA overexpression alleviated lipid metabolism in a BDH1-dependent manner in HFD-fed mice. a A scheme for HFD feeding and HADHA interference. Eight-week-old mice injected with AAV-NC, AAV-HADHA or AAV-BDH1 shRNA were fed with NCD or HFD for 12 weeks. **b, c** mRNA levels of *Hadha* and *Bdh1* in the liver, heart, kidney and muscle of mice before HFD feeding (n = 3). **d** Western blotting analysis of BDH1 in the liver of mice injected with AAV-NC or AAV-BDH1 shRNA. It was repeated 3 times independently with similar results. **e** Weight gain and food intake (n = 6). **f** Epididymal fat pad. AAV adeno-associated virus, HFD high-fat diet, NC normal control, NCD normal chow diet, OE overexpression, KD knockdown. Values represent mean ± SEM. Statistical differences were determined by two-tailed Student's *t* test. Source data are provided as a Source Data file.

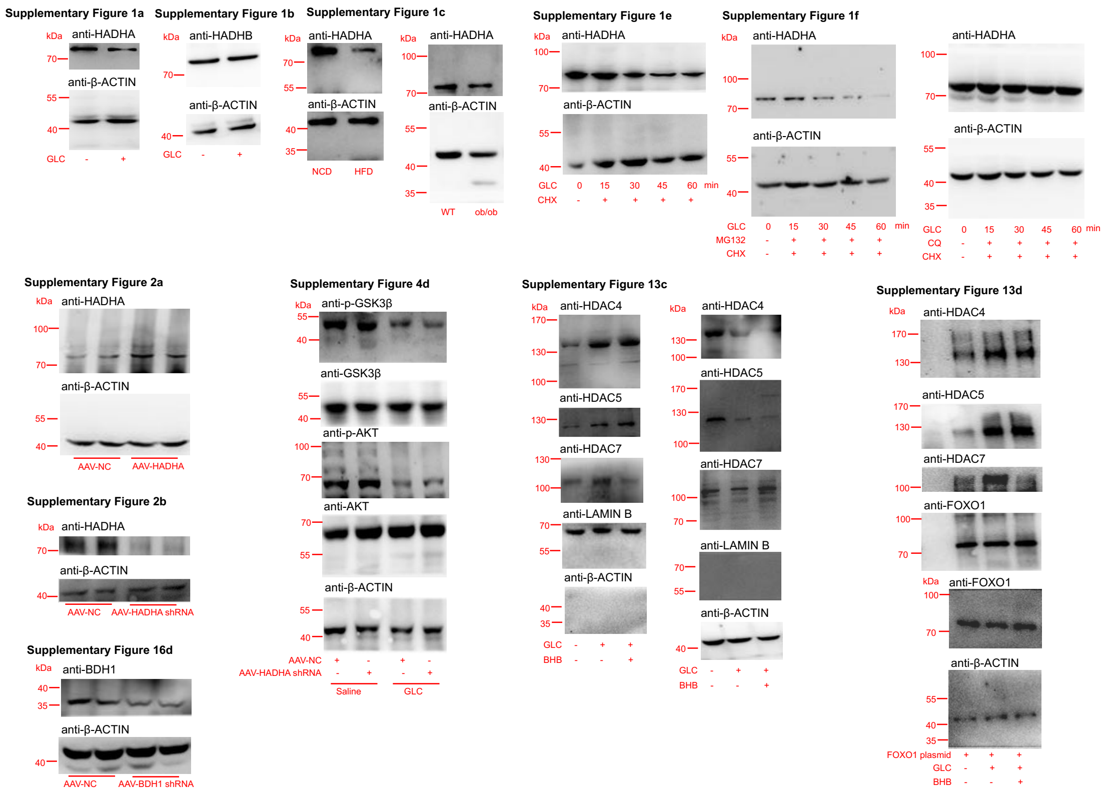

Supplementary Fig. 17 Original source date of the blotting images.

Supplementary Table 1. Primer pairs for qRT-PCR.

Gene		Sequence 5’-3’
Hadha (mice)	Forward	TGCATTTGCCGCAGCTTTAC
	Reverse	AAGCATTCAACGCCAGGTTC
Pck1 (mice)	Forward	TGGCATCCCCGAATATGATGA
	Reverse	GGGCGAGTCTGTCAGTTCAAT
G6pc (mice)	Forward	CGACTCGCTATCTCCAAGTGA
	Reverse	GTTGAACCAGTCTCCGACCA
Pgc1a (mice)	Forward	ATACCGCAAAGAGCACGAGAAG
	Reverse	CTCAAGAGCAGCGAAAGCGTCACAG
Bdh1 (mice)	Forward	TGACACCCGTCGGACCTAC
	Reverse	TTCTCAGTCGGTCACTCTTCA
Hdac7 (mice)	Forward	GGCAGGCTTACACCAGCAA
	Reverse	TGGGCAGGCTGTAGGGAATA
Actb (mice)	Forward	AGTGTGACGTTGACATCCGTA
	Reverse	GCCAGAGCAGTAATCTCCTTCT

Supplementary Table 2. Antibodies for immunoblotting.

Antibody	Cat No.	Company	Dilutions	Source
HADHA	sc-374497	Santa Cruz	1:400	Mouse
HADHB	ab110301	Abcam	1:1000	Rabbit
FOXO1	2880s	CST	1:1000	Rabbit
p-FOXO1	9461s	CST	1:1000	Rabbit
Acetylated-Lysine	9441s	CST	1:1000	Rabbit
HDAC4	sc-46672	Santa Cruz	1:400	Mouse
HDAC5	sc-133225	Santa Cruz	1:400	Mouse
HDAC7	sc-74563	Santa Cruz	1:400	Mouse
LAMIN B	17416s	CST	1:1000	Rabbit
AKT	4691s	CST	1:1000	Rabbit
p-AKT	4060s	CST	1:1000	Rabbit
GSK3β	5676s	CST	1:1000	Rabbit
p-GSK3β	9322s	CST	1:1000	Rabbit
BDH1	ab193156	Abcam	1:1000	Rabbit
β-ACTIN	bs-0061R	Bioss	1:500	Rabbit
Mouse Anti-Rabbit IgG	93702s	CST	1:1000	
Normal Rabbit IgG	2729s	CST	1:1000	
