# Supplementary material for: The mitochondrial β-oxidation enzyme HADHA restrains hepatic glucagon response by promoting β-hydroxybutyrate production
Source: Nat Commun. 2022 Jan 19;13:386. doi: 10.1038/s41467-022-28044-x (PMC8770464; doi:10.1038/s41467-022-28044-x)
Supplement: Supplementary file 3 — Description of Additional Supplementary file [file 41467_2022_28044_MOESM3_ESM.docx]

**Description of Additional Supplementary file**

File Name: Supplemental Data 1
Description: Multiple reaction monitoring chromatograms of β-hydroxybutyrate, acetoacetate, citrate and acetyl-CoA in primary hepatocytes by ^13^C stable isotope tracing. Hepatocytes were treated with [U-^13^C]palmitate (0.1 mM) for 4 h followed by 100 nM glucagon stimulation for 1 h with or without HADHA plasmid/siRNA transfection. Both labeled (M2, M4) and unlabeled (M0) metabolite fractions were detected.
