# Supplementary figures and images for: The mitochondrial β-oxidation enzyme HADHA restrains hepatic glucagon response by promoting β-hydroxybutyrate production
Source: Nat Commun. 2022 Jan 19;13:386. doi: 10.1038/s41467-022-28044-x (PMC8770464; doi:10.1038/s41467-022-28044-x)

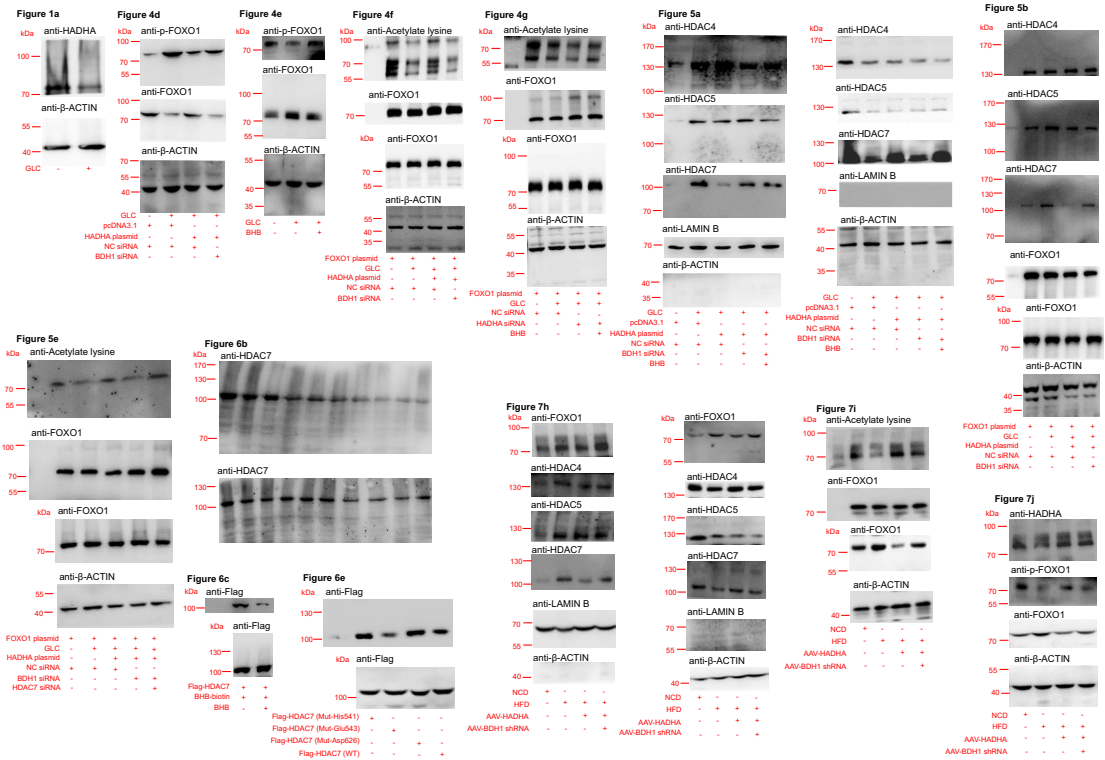

Original source date of the blotting images.

Supplement: Supplementary file 6 — Source Data [file 41467_2022_28044_MOESM6_ESM.zip › Soure Data/ Source Date of Blotting Images.pdf]
